# Clinical and Prognostic Value of PET/CT Imaging with Combination of ^68^Ga-DOTATATE and ^18^F-FDG in Gastroenteropancreatic Neuroendocrine Neoplasms

**DOI:** 10.1155/2018/2340389

**Published:** 2018-02-26

**Authors:** Panpan Zhang, Jiangyuan Yu, Jie Li, Lin Shen, Nan Li, Hua Zhu, Shizhen Zhai, Yan Zhang, Zhi Yang, Ming Lu

**Affiliations:** ^1^Department of Gastrointestinal Oncology, Key Laboratory of Carcinogenesis and Translational Research, Ministry of Education, Peking University School of Oncology, Beijing Cancer Hospital and Institute, Beijing, China; ^2^Department of Nuclear Medicine, Key Laboratory of Carcinogenesis and Translational Research, Ministry of Education, Peking University School of Oncology, Beijing Cancer Hospital and Institute, Beijing, China

## Abstract

**Background:**

To evaluate the clinical and prognostic value of PET/CT with combination of ^68^Ga-DOTATATE and ^18^F-FDG in gastroenteropancreatic neuroendocrine neoplasms (GEP-NENs).

**Method:**

83 patients of GEP-NENs who underwent ^68^Ga-DOTATATE and ^18^F-FDG PET/CT were enrolled between June 2013 and December 2016. Well-differentiated (WD) NETs are divided into group A (Ki-67 < 10%) and group B (Ki-67 ≥ 10%), and poorly differentiated (PD) NECs are defined as group C. The relationship between PET/CT results and clinicopathological characteristics was retrospectively investigated.

**Result:**

For groups A/B/C, the sensitivities of ^68^Ga-DOTATATE and ^18^F-FDG were 78.8%/83.3%/37.5% and 52.0%/72.2%/100.0%. A negative correlation between Ki-67 and SUV_max_ of ^68^Ga-DOTATATE (*R* = −0.415; *P* ≤ 0.001) was observed, while a positive correlation was noted between Ki-67 and SUV_max_ of ^18^F-FDG (*R* = 0.683; *P* ≤ 0.001). 62.5% (5/8) of patients showed significantly more lesions in the bone if ^68^Ga-DOTATATE was used, and 22.7% (5/22) of patients showed more lymph node metastases if ^18^F-FDG was used.

**Conclusions:**

The sensitivity of dual tracers was correlated with cell differentiation, and a correlation between Ki-67 and both SUV_max_ of PET-CTs could be observed. ^68^Ga-DOTATATE is suggested for WD-NET and ^18^F-FDG is probably suitable for patients with Ki-67 ≥ 10%.

## 1. Introduction

Gastroenteropancreatic neuroendocrine neoplasms (GEP-NENs) are a heterogeneous group of neoplasms that arise from cells of the endocrine system [1]. GEP-NENs are rare and present many clinical challenges. Because of their unpredictable biologic behaviors, the diagnosis usually takes place only after the condition has become advanced. Treatment regimens rely mainly on histological grading via biopsy; however, tumor heterogeneity cannot be fully assessed by tumor biopsy [2, 3]. Although Ki-67 staining has been shown to have prognostic significance in GEP-NENs, pitfalls may lead clinicians to misjudge the tumor grades. Specifically, first, the current gold-standard method, in which 2000 cells are counted, is heavily dependent on the skill and expertise of the reporting pathologist [4]. Second, the limited tissue in some cases may impede accurate assessment of Ki-67 given the potential for heterogeneity of Ki-67 expression within tumors. Finally, the Ki-67 index may vary over time in the same patient, with changes possible both in response to treatment [5] and over the progression of the disease [6]. We believe that combined dual-tracer PET/CT imaging offers distinct advantages to overcome the above pitfalls.

DOTA-peptides specifically bind to somatostatin receptors 2, 3, and 5 and are usually overexpressed on the surfaces neuroendocrine cells [7, 8]. ^68^Ga-DOTATATE has been shown to be useful for staging, restaging, surveillance, determining SSTR-based therapy, and monitoring responses to treatments in NENs [9, 10]. ^18^F-FDG is a glucose analogue, and PET/CT imaging with this tracer has been shown to be correlated with NENs aggressiveness. The presence of increased glucose in NENs highlights an increased propensity for invasion and metastasis, and PET imaging with ^18^F-FDG accordingly has higher sensitivity, especially in aggressive and high-grade tumors [11, 12]. Therefore, we believe that PET/CT imaging with combination of  ^68^Ga-DOTATATE and ^18^F-FDG PET/CT is a highly efficient whole-body imaging method, and it could be complementary to conventional imaging methods.

A large number of previous studies have evaluated the diagnostic accuracy of both tracers in the presence of a relative shortage of information, regarding the correlation to pathological findings and prognostic value. Only a few studies have compared the clinical impact of both ^68^Ga-DOTATATE and ^18^F-FDG PET tracers on NENs [13, 14]. The present study aimed to determine the clinical value of the complementary PET/CT imaging method in a large histologically proven NENs population.

## 2. Materials and Methods

We analyzed the data from 83 (50 males and 33 females) consecutive patients with pathologically proven NENs who underwent contemporaneous PET/CT imaging with ^68^Ga-DOTATATE and ^18^F-FDG between June 2013 and December 2016. ^68^Ga-DOTATATE and ^18^F-FDG PET/CT scans were performed within an interval of no more than 2 weeks. No patients were treated during this interval. All NENs were classified according to the histopathological reports, which are based on recent consensus statements of the European Neuroendocrine Tumor Society. According to the grade of differentiation, proliferation index (Ki-67), and mitotic count, the well-differentiated (WD) neoplasms are herein defined as NET and graded G1 (Ki-67 ≤ 2%) or G2 (Ki-67 3–20%) and G3a (Ki-67 > 20%); the poorly differentiated (PD) neoplasms are defined as NEC and G3b. G2 patients were further divided into 2 groups as G2a (3–9%) and G2b (10–20%).

### 2.1. PET/CT Acquisition

Patients fasted for at least 6 h before PET/CT scan. Images were acquired 1 h after injection of 3.7 MBq/kg ^18^F-FDG or 1 h after the injection of 100–200 MBq ^68^Ga-DOTATATE. Whole-body scan (brain to mid-thigh) was performed with the patient in the supine position. CT exposure factors for all scans were 120 kV and 100 mA. PET/CT images were reported in consensus by two experienced nuclear medicine physicians who were blinded to the findings of the structural imaging. Any nonphysiological focus of ^68^Ga-DOTATATE or ^18^F-FDG uptake greater than the normal liver background was considered positive. At the same time, CT imaging was used to differentiate between lesions and physiological uptake. The maximum standardized uptake value (SUV_max_) of primary and metastatic lesions was calculated. SUV_max_ generated from each patient was used in the final analysis. The SUV ratio of the tumor relative to the maximal liver uptake was calculated by dividing SUV_max_ of the tumor by SUV_max_ of the liver (SUV_T/L_). The ratio between SUV_max_ of  ^68^Ga-DOTATATE and that of ^18^F-FDG (SUV_max_ ratio) was also calculated.

### 2.2. Statistical Analysis

Descriptive analyses are presented using mean and SD for normally distributed variables, but median, minimum, and maximum values were used for those that were nonnormally distributed. Analyses were performed using SPSS (version 21.0; IBM). The paired Student's *t*-test was used for the related nonnormally distributed variables. Correlation of SUV_max_ values of ^68^Ga-DOTATATE and ^18^F-FDG with Ki-67 index was assessed using Spearman's correlation coefficient. Overall survival (OS) was calculated from the date of diagnosis to the last day of follow-up or death. Kaplan-Meier survival analysis was performed to assess the prognostic value regarding OS, and differences between groups were analyzed using the log-rank test. *P* < 0.05 was considered as statistical significance.

## 3. Results

### 3.1. Patient Characteristics

A total of 83 (50 males and 33 females) patients were included in the study, with a median age of 56 years (range: 27–77 years). The primary tumors were located in the pancreas in 27 patients (32.5%), gastrointestinal tract in 43 patients (51.8%), and unknown locations in 13 patients (15.7%). Among the subjects, 48 patients (57.8%) had lymph node involvement and 60 patients (72.3%) had distant metastases. Pathological evaluation showed that 51 patients (61.4%) had WD-NET and 32 patients (38.6%) had PD-NEC. Among WD-NETs, there were 14 patients (16.9%) in G1 (≤2%), 19 patients (22.9%) in G2a (3–9%), 9 patients (10.8%) in G2b (10–20%), and 28 patients (33.7%) in G3a (>20%) (Table 1).

### 3.2. Complementary PET/CT Qualitative Evaluation

#### 3.2.1. Comparison in Sensitivity

For all patients, ^68^Ga-DOTATATE was positive in 53 cases and negative in 30 cases, while ^18^F-FDG PET/CT was positive in 62 cases and negative in 21 cases. Overall, ^18^F-FDG assessment was found to have a better sensitivity (74.7%) compared with ^68^Ga-DOTATATE (63.8%), although it is not statistically significant (*P* = 0.593). Remarkably, we found that the sensitivity of dual tracers (94.0%) was significantly higher than that with ^68^Ga-DOTATATE or ^18^F-FDG alone (*P* < 0.01). For NET and NEC, the sensitivity was 80.39% (41/51) and 37.5% (12/32) with ^68^Ga-DOTATATE and was 58.82% (30/51) and 100% (32/32) with ^18^F-FDG. Separating from the primary sites, the sensitivity of ^68^Ga-DOTATATE in pancreatic NET was higher than that in gastrointestinal NET (NET: 89.5% versus 75.0%, *P* = 0.034; NEC: 75.0% versus 31.6%, *P* = 0.027). However, ^18^F-FDG showed no difference between pancreatic and gastrointestinal NENs in terms of sensitivity.

#### 3.2.2. Complementary PET/CT Semiquantitative Evaluation

**Figure 1:**
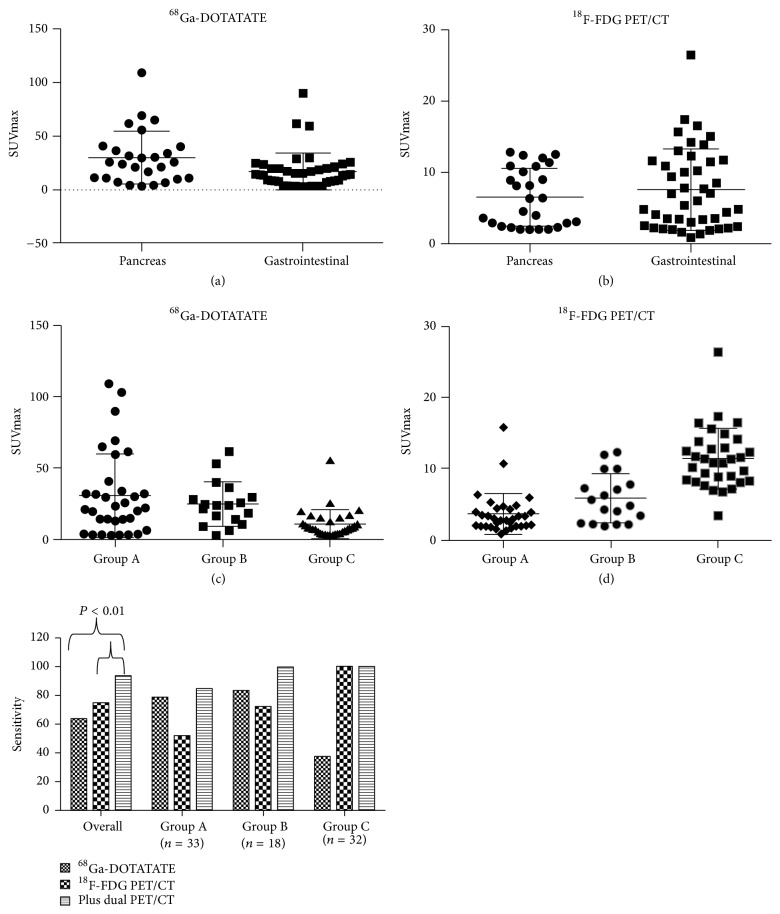
Comparison of SUV_max_ of PET/CT according to primary sites ((a) and (b)) and tumor grade ((c) and (d)). The sensitivity of group A (G1 + G2a = Ki-67 < 10%), group B (G2b + G3a = well-differentiated neoplasms with Ki-67 ≥ 10%), and group C (G3b = poorly differentiated neoplasms with Ki-67 > 20%) in PET/CT imaging.

Table 2 illustrates the sensitivities and SUV_max_ in correlation with primary sites and grades. The uptake values of ^68^Ga-DOTATATE in PanNENs were significantly higher than those in GI-NEN (29.87 ± 4.78 versus 16.76 ± 2.62, *P* = 0.011). The values of ^18^F-FDG PET/CT in PanNEN had a trend toward a lower SUV than that in GI-NEN (6.51 ± 0.77 versus 7.57 ± 0.86, *P* = 0.067) (Figure 1).

#### 3.2.3. Comparison in Different Pathological Groups

The sensitivity of ^68^Ga-DOTATATE and ^18^F-FDG in G1/G2a/G2b/G3a/G3b was 78.6%/73.3%/88.9%/77.8%/37.5% and 50.0%/52.6%/66.7%/77.8%/100.0%. ^68^Ga-DOTATATE imaging provided a sensitivity of >73% in WD-NET (G1–G3a) and only 37.5% in PD-NEC (G3b). For G1 and G2a (Ki-67 < 10%), a statistically significant positive correlation between Ki-67 and the sensitivity with ^18^F-FDG could be found, and the sensitivity in G1 and G2a (Ki-67 < 10%) was about 50%, increasing dramatically when the Ki-67 index was over 10%. The sensitivity in G3a reached 77.8% and that in G3b was 100%. The patients were divided into 3 groups: group A (G1 + G2a), group B (G2b + G3a), and group C (G3b). With this grouping, the sensitivity of ^68^Ga-DOTATATE and ^18^F-FDG in group A/B/C was 78.8%/83.3%/37.5% and 52.0%/72.2%/100.0%. Importantly, we found that the sensitivities of imaging with dual tracers in groups A/B/C were 84.8%/100%/100%, which were significantly higher than that with the single tracer. There was a significant negative correlation between Ki-67 and ^68^Ga DOTATATE SUV_max_ (*R* = − 0.415; *P* ≤ 0.001), while a positive correlation was noted between Ki-67 and ^18^F-FDG SUV_max_ value (*R* = 0.683; *P* ≤ 0.001). Moreover, ^68^Ga-DOTATATE SUV_T/L_ showed a negative correlation with Ki-67 index (*R* = − 0.357; *P* = 0.001). However, ^18^F-FDG SUV_T/L_ was positively correlated with Ki-67 index (*R* = 0.617; *P* ≤ 0.001) (Figure 1).

#### 3.2.4. Concordant and Discordant Findings

When combining the results of the dual-tracer PET/CT, 37 patients were positive in both tracers, and 16 patients were ^68^Ga-DOTATATE-positive and ^18^F-FDG-negative, while 25 patients were ^18^F-FDG-positive and ^68^Ga-DOTATATE-negative. 5 patients were negative in both tracers (Table 3).

**Figure 2:**
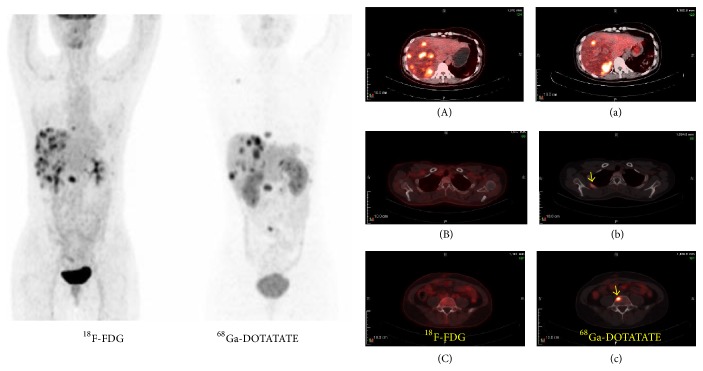
A 37-year-old women with pancreatic NEC G3 (Ki-67 = 80%) and lymph node, liver, and bone metastases, from whom the primary lesion has been resected.^ 18^F-FDG PET/CT showed more liver lesions, while ^68^Ga-DOTATATE detected more bone lesions. ((A) and (a)) Liver lesions showed heterogeneity in SSTR expression. ((B) and (b) and (C) and (c)) ^18^F-FDG PET/CT failed to show bone metastases in rib and lumbar vertebra.

^68^Ga-DOTATATE and ^18^F-FDG PET/CT findings were concordant in 37 patients with 25 WD-NETs and in 12 PD-NECs. Only 1 patient was diagnosed as localized duodenal NEN. The remaining 36 patients had regional lymph node metastasis, distant metastasis, or both mainly occurring in liver or bone. Of 27 patients with liver metastases, 8 patients (29.6%) examined via ^68^Ga-DOTATATE showed heterogeneity in SSTR expression. Of 8 patients with bone metastases, imaging findings of 5 patients (62.5%) demonstrated that ^68^Ga-DOTATATE highlighted more bone lesions than ^18^F-FDG PET/CT. Of 22 patients with lymph node involvement, the dual tracers with 5 patients (22.7%) showed that ^18^F-FDG findings could be more prominent than those with ^68^Ga-DOTATATE (Figure 2).

5 patients showed negative results in both two tracers, with a histological diagnosis of group A. 1 patient was diagnosed as duodenal NET G2 (Ki-67: 3%) with multiple liver metastases (largest lesion: 7.2 cm × 4.7 cm). The remaining 4 patients had rectal or gastric NET (G1/2) with lesions smaller than 5 mm.

### 3.3. Treatment and Follow-Up

**Figure 3:**
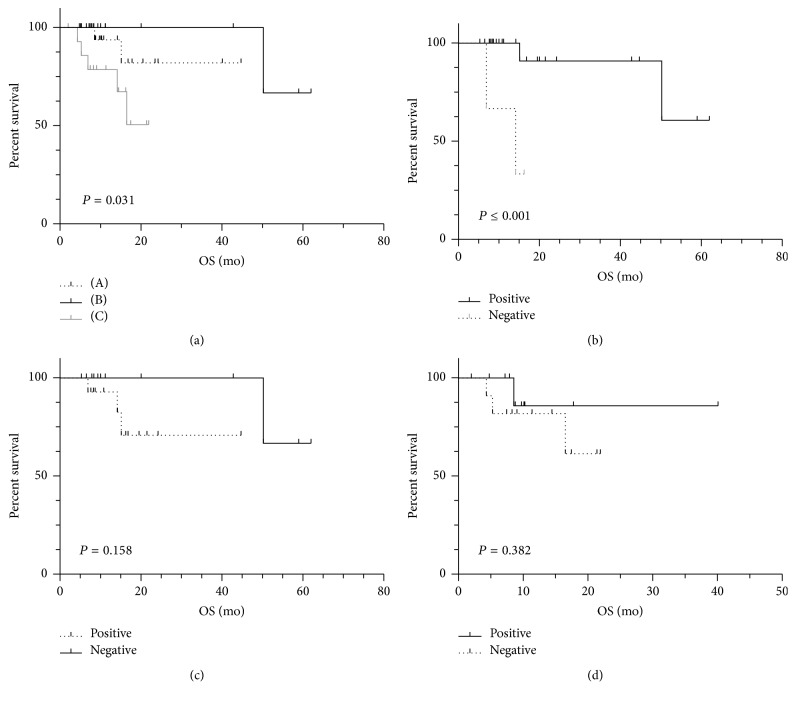
The overall survival of unresectable patients detected with dual tracers: (a) Kaplan-Meier survival curve for unresectable patients, (A) positive for both tracers, (B) ^68^Ga-DOTA-TATE only, and (C) ^18^F-FDG only; (b) unresectable NET patients with ^68^Ga-DOTA-TATE results (positive or negative); (c) unresectable NET patients with ^18^F-FDG results (positive or negative); (d) unresectable NEC patients with ^68^Ga-DOTA-TATE results (positive or negative).

Of the 83 patients, 26 performed radical surgery; 57 (31 NET and 26 NEC) unresectable patients were treated with palliative surgery, SSA, chemotherapy, and TACE. The median follow-up was 21 months (in the range of 2–62 months). During the follow-up period, 9 (6 NEC and 3 NET) patients died of the progressive disease. Unresectable patients with positive results solely with ^18^F-FDG showed the worst prognosis, while those positive solely with ^68^Ga-DOTATATE showed the best prognosis. For unresectable patients with NET (*n* = 31), being ^68^Ga-DOTATATE-negative was associated with worse prognosis (HR: 10.4; 95% CI: 1.5–78.2; *P* ≤ 0.001), and being ^18^F-FDG-positive tended to be correlated with a worse prognosis (HR: 3.6; 95% CI: 0.7–9.8; *P* = 0.158). For unresectable patients with NEC (*n* = 26), being ^68^Ga-DOTATATE-negative also tended to be associated with a worse prognosis (HR: 2.4; 95% CI: 0.3–5.4; *P* = 0.382) (Figure 3).

## 4. Discussion

^68^Ga-DOTATATE and ^18^F-FDG PET/CT play a crucial role in the diagnosis and clinical management of NENs with morphologic and functional information. ^68^Ga-DOTATATE was found to be superior to ^18^F-FDG in WD-NET, whereas ^18^F-FDG was more sensitive in PD-NEC [15]. Considering the costs of molecular imaging, choosing the selected patients for the specific PET/CT imaging is of vital importance. Analyzing the results produced using both tracers for different grades and primary sites could be a balanced approach. The aim of the present study was to compare ^68^Ga-DOTATATE and ^18^F-FDG PET/CT in GEP-NENs and to investigate the relationship between the complementary PET/CT results and histopathologic findings in clinical and prognostic values in a large, histologically confirmed NEN population.

^68^Ga-DOTATATE imaging and ^18^F-FDG PET/CT imaging have been compared in several studies which have been shown to have variable sensitivities in detecting NENs with a relatively small number of patients. Naswa et al. [16] reported that the sensitivity of ^68^Ga-DOTANOC and ^18^F-FDG was 91.4% and 42.5%, respectively. Koukouraki et al. [17] demonstrated that the sensitivity of ^68^Ga-DOTATOC and ^18^F-FDG was 90% and 68%, respectively, and ^68^Ga-DOTANOC was more sensitive in the detection of primary sites or metastasis than ^18^F-FDG [18]. Notably the patients included in this study were mainly WD-NETs with lower glucose metabolism. In the present study, the sensitivity of ^68^Ga-DOTATATE and ^18^F-FDG PET/CT was 63.85% and 74.70%, respectively. Subgroup analysis showed that the sensitivity of ^68^Ga-DOTATATE was mainly correlated with the degree of differentiation, instead of correlation with Ki-67 index. The sensitivity of ^18^F-FDG showed a positive correlation with the Ki-67 index and differentiation. From ^18^F-FDG imaging, the sensitivity was under 53% for patients with Ki-67 < 10% and 100% for PD-NEC. SUV_max_ of patients with NEC was low even for those who had positive results under ^68^Ga-DOTATATE. We also observed that patients with Ki-67 < 10% showed low uptake in ^18^F-FDG PET/CT. On consideration of the weak significance of ^68^Ga-DOTATATE for PD-NEC and ^18^F-FDG PET/CT for lower-grade NET, our study demonstrated that ^18^F-FDG is more suitable for patients with Ki-67 ≥ 10%, and ^68^Ga-DOTATATE is less advantageous in PD-NEC and should be tailored to the individual patients.

Our study demonstrated that PET/CT uptake was statistically significantly different between subgroups of GEP-NENs according to grading. The cohort was separated into 3 groups: A (G1 + G2a), B (G2b + G3a), and C (G3b). Group B showed higher sensitivity for ^18^F-FDG than group A, and the median SUV_max_ increased significantly, indicating relatively quick proliferation rate associated with Ki-67 index. Strosberg [19] proposed chemotherapy as a treatment option for tumors with Ki-67 ≥ 10%, especially those with higher ^18^F-FDG activity. Such tumors showed high proliferative capacity and aggressive behavior and are recommended for chemotherapy after PRRT or somatostatin therapy. In this way, ^18^F-FDG PET/CT may be suitable for identifying patients with aggressive conditions in group B who could benefit from chemotherapy. Thus, PET/CT imaging may establish the missing link between histopathologic findings and the treatment regimen.

**Figure 4:**
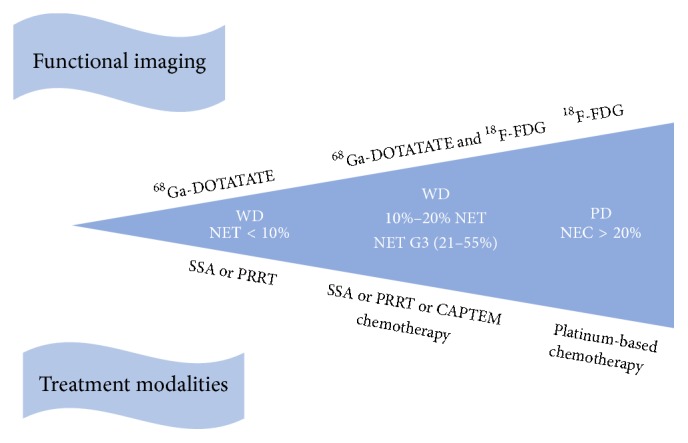
PET/CT imaging and treatment regime.

Based on our results, dual tracers' assessment is recommended for WD-NET with Ki-67 ≥ 10% (G2b and G3a). ^68^Ga-DOTATATE and ^18^F-FDG were complementary in detecting lesions and dual-trace PET/CT showed an advantage in the assessment of SSA, PRRT, and chemotherapy. We proposed that WD-NET patients with Ki-67 ≥ 10% should be examined using dual tracers upon diagnosis. We also suggest that ^68^Ga-DOTATATE should be performed solely in WD-NET patients with Ki-67 < 10% and ^18^F-FDG is sufficient for PD NEC. Moreover, repeated PET/CT is warranted when disease progresses rapidly, considering the heterogeneous expression and complementary findings to histopathology (Figure 4).

We investigated the correlations between dual tracers and Ki-67 index. SUV_max_ or SUV_T/L_ was positively correlated with Ki-67 index with respect to ^18^F-FDG PET/CT and negatively correlated with Ki-67 index with respect to ^68^Ga-DOTATATE. SUV_max_ of PET/CT may be a suitable biomarker for evaluation of the biological behavior of NENs. The relationship between SUV_T/L_ and Ki-67 index was weak, but it showed a consistency with SUV_max_. However, SUV_max_ may be influenced by technical elements, while SUV_T/L_ could reduce the differences attributable to equipment and operation to some extent.

GEP-NENs are a heterogeneous group of neoplasms that display great variability in biological behaviors and clinical outcomes [20]. This requires accurate diagnostic techniques for precise staging and choice of therapy. The standard grading is mainly based on the immunohistochemistry of the proliferation marker Ki-67. However, there are many lesions with variable tracer uptake at different parts of the tumor. Especially within the same organ, these lesions may prevent the biopsy from being a comprehensive reflection of tumor heterogeneity in vivo, leading to inaccurate Ki-67 index results. Therefore, dual-trace PET/CT, which is a whole-body noninvasive alternative, is warranted to overcome the shortcomings of histopathologic grading. The findings of this study included the ability of ^68^Ga-DOTATATE to detect heterogeneity in tumors and variable expression in primary sites. SUV_max_ of PanNEN was higher than that of GI-NENs, which was consistent with prior studies [21, 22], where the investigators found higher levels of messenger RNA expression of SSTR2 and SSTR5 in pancreatic than in gastrointestinal NENs. In this way, ^68^Ga-DOTATATE is more sensitive in PanNEN, but it may miss some lesions in GI-NEN. However, SUV_max_ of PanNEN and GI-NEN showed no difference for ^18^F-FDG, which is complementary to ^68^Ga DOTATATE. Another finding of the present study was that ^68^Ga DOTATATE has a superb ability to detect heterogeneity in metastatic lesions, which is beneficial for us to select the optimal protocol. We believe that complementary PET/CT can evaluate the tumors' heterogeneity and influence treatment options.

The morphological findings and Ki-67 index are considered important prognostic markers in NENs. One major limitation of these histopathological parameters as prognostic markers is the requirement for tissue sampling, which is not always feasible. A few studies have demonstrated the prognostic value of ^18^F-FDG and ^68^Ga-DOTATATE PET/CT in patients with NENs. ^18^F-FDG was an independent predictor of PFS [23, 24]. ^18^F-FDG SUV_max_ > 3 was demonstrated to be independent predictor of disease progression, superior to conventional prognostic factors such as Ki-67 index and serum CgA [25]. SSTR expression was found to be a positive prognostic factor, and it was therefore expected that SSTR-base PET/CT would also have prognostic value in NENs [26]. Sharma et al. [27] demonstrated the prognostic value of SUV_max_ measured with ^68^Ga DOTATATE in 37 patients with NET. SUV_max_ ≥ 14.5 was found to distinguish patients with progressive and those with nonprogressive disease. A major limitation of the prior studies was that most of the patients included were WD-NET, which may involve selection bias. Our study enrolled 57 unresectable patients (31 NET and 26 NEC). Unresectable patients positive with ^18^F-FDG alone showed the worst prognosis, while those positive with ^68^Ga-DOTATATE alone had the best prognosis. NET patients with predominant ^18^F-FDG uptake and a negative ^68^Ga-DOTATATE scans had worse prognosis. There is a strong association between high ^18^F-FDG uptake and worse outcome even in patients with WD-NETs. However, PD-NEC with negative ^68^Ga-DOTATATE uptake may lead to worse prognosis. The present study has several limitations. First, our study is retrospective, and the second limitation is that the follow-up time was not long enough to assess treatment response, considering the relatively inert biological behavior of NENs.

Noninvasive dual-tracer imaging with ^68^Ga-DOTATATE and ^18^F-FDG PET/CT seems promising as an alternative to tissue sampling, due to its capacity to reflect two different aspects of tumor biology, SSTR expression and glucose metabolism, respectively. Accordingly, imaging with dual tracer is recommended for WD-NET patients with Ki-67 ≥ 10%, providing information for selection of SSA, PRRT, and chemotherapy. Taking the advantages of dual-tracer imaging in detecting lesions is useful for accurate clinical management. Dual-tracer imaging also shows a clearly linear correlation between SUV_max_ and Ki-67 index. On consideration of heterogeneous expression and complementary findings to histopathology, our results suggested that repeated dual-tracer imaging is warranted to evaluate dynamic biological behavior and prognosis.

## Figures and Tables

**Table 1:** Patient characteristics.

Characteristics	NET (%)	NEC (%)	All (%)
Gender			
Female (*n*)	26 (51.0)	7 (21.9)	33 (39.7)
Male (*n*)	25 (49.0)	25 (78.1)	50 (60.3)
Primary sites (*n*)			
Pancreas	19 (37.3)	8 (25.0)	27 (32.5)
Gastrointestinal	24 (47.0)	19 (59.4)	43 (51.8)
Primary unknown	8 (15.7)	5 (15.6)	13 (15.7)
Metastatic sites (*n*)			
Liver	36 (70.6)	13 (40.6)	49 (59.0)
Lymph nodes	21 (41.2)	27 (84.4)	48 (57.8)
Bone	9 (17.6)	10 (31.3)	19 (22.9)
Lung	4 (7.8)	1 (3.1)	5 (6.0)
Other	6 (11.7)	7 (21.9)	13 (15.7)

NET, neuroendocrine tumor; NEC, neuroendocrine carcinoma.

**Table 2:** Sensitivity and uptake of ^68^Ga-DOTATATE and ^18^F-FDG PET/CT for different primary sites and grades.

	Sensitivity (%)		SUV_max_ (mean ± SD)	
	^68^Ga-DOTATATE	^18^F-FDG PET/CT	^68^Ga-DOTATATE	^18^F-FDG PET/CT
Primary lesion				
Gastrointestinal tract	55.8%	74.4%	16.75 ± 2.62	7.56 ± 0.87
Pancreas	85.2%	66.7%	29.87 ± 4.77	6.51 ± 0.78
WD NET	80.4%	58.8%	28.87 ± 3.52	4.51 ± 0.45
Gastrointestinal NET	75.0%	54.2%	22.68 ± 2.77	3.71 ± 0.45
Pancreatic NET	89.5%	52.6%	31.19 ± 4.25	5.13 ± 0.93
PD NEC	37.5%	100.0%	10.86 ± 1.78	11.46 ± 0.75
Gastrointestinal NEC	31.6%	100.0%	9.26 ± 1.25	12.44 ± 1.11
Pancreatic NEC	75.0%	100.0%	18.23 ± 5.93	10.23 ± 0.67

WD, well-differentiated; PD, poorly differentiated; NET, neuroendocrine tumor; NEC, neuroendocrine carcinoma.

**Table 3:** Concordant and discordant findings.

^68^Ga-DOTATATE	Positive	Positive	Negative	Negative
^18^F-FDG PET/CT	Positive	Negative	Positive	Negative
Primary lesion (*n*)				
Pancreas	14	9	4	0
Gastrointestinal tract	18	6	14	5
CUP	5	1	7	0
Metastatic sites				
Liver	27	11	9	1
Lymph node	22	7	18	0
Bone	8	2	8	0
Ki-67 range				
Group A (*n* = 33)	15 (45.5%)	11 (33.3%)	2 (6.1%)	5 (15.1%)
Group B (*n* = 18)	10 (55.5%)	5 (27.8%)	3 (16.7%)	0
Group C (*n* = 32)	12 (37.5%)	0	20 (62.5%)	0

CUP, Cancer of unknown primary.

## Abbreviations

GEP-NENs: Gastroenteropancreatic neoplasms
PD: Poorly differentiated
WD: Well-differentiated
OS: Overall survival
NET: Neuroendocrine tumor
NEC: Neuroendocrine carcinoma
PRRT: Peptide receptor radionuclide therapy.
